# Population-based real-world registry study to evaluate clinical outcomes of chronic graft-versus-host disease

**DOI:** 10.1371/journal.pone.0282753

**Published:** 2023-03-09

**Authors:** Igor Novitzky-Basso, Frida Schain, Nurgul Batyrbekova, Thomas Webb, Mats Remberger, Armand Keating, Jonas Mattsson

**Affiliations:** 1 Princess Margaret Cancer Centre, Toronto, Canada; 2 Department of Medicine, University of Toronto, Toronto, Canada; 3 Janssen Global Services, Stockholm, Sweden; 4 Schain Research AB, Bromma, Sweden; 5 Department for Medicine, Division of Hematology, Karolinska Institutet, Stockholm, Sweden; 6 SDS Life Science, Stockholm, Sweden; 7 Department of Medical Epidemiology and Biostatistics, Karolinska Institutet, Stockholm, Sweden; 8 Janssen Global Services, High Wycombe, United Kingdom; 9 Department of Medical Sciences, Uppsala University and Clinical Research and Development Unit, Uppsala University Hospital, Uppsala, Sweden; 10 Gloria and Seymour Epstein Chair in Cell Therapy and Transplantation, Department of Medicine, University of Toronto, Toronto, Canada; 11 Department of Oncology-Pathology, Karolinska Institutet, Stockholm, Sweden; Istanbul University-Cerrahpaşa, Cerrahpaşa Faculty of Medicine, TURKEY

## Abstract

**Introduction:**

Chronic graft-versus-host disease (cGVHD) is a serious immune-mediated complication after allogeneic haematopoietic stem cell transplantation (HSCT), but in patients with malignancy, cGVHD development is associated with superior survival. Lack of reliable biomarkers and clinical underreporting means there is insufficient understanding of cGVHD clinical outcomes and balance between cGVHD treatment and maintaining beneficial graft-versus-tumour effects.

**Methods:**

We performed a Swedish population-wide registry study following patients who underwent allogeneic HSCT 2006–2015. cGVHD status was retrospectively classified using a real-world method based on the timing and extent of systemic immunosuppressive treatment.

**Results:**

cGVHD incidence among patients surviving ≥6 months post-HSCT (n = 1246) was 71.9%, significantly higher than previously reported. 5-year overall survival in patients surviving ≥6 months post-HSCT was 67.7%, 63.3%, and 65.3%, in non-, mild, and moderate-severe cGVHD, respectively. Non-cGVHD patients had a mortality risk almost five-fold higher compared to moderate-severe cGVHD patients 12-months post-HSCT. Moderate-severe cGVHD patients had greater healthcare utilization compared with mild and non cGVHD patients.

**Conclusion:**

cGVHD incidence was high among HSCT survivors. Non-cGVHD patients had higher mortality during the first 6 months of follow-up; however, moderate-severe cGVHD patients had more comorbidities and healthcare utilization. This study highlights the urgent need for new treatments and real-time methods to monitor effective immunosuppression after HSCT.

## Introduction

Successful allogeneic haematopoietic stem cell transplantation (HSCT) relies on optimizing the balance between graft-versus-host disease and the graft-versus-leukaemia effect to mitigate the risk of disease relapse. Chronic graft-versus-host disease (cGVHD) is a severe complication of HSCT and a leading cause of morbidities following HSCT [1, 2]. The pathophysiology of cGVHD includes complex interactions between different immune cells from the recipient and the donor, yet cGVHD may contribute to prolonging post-relapse survival in patients with haematological malignancies [3, 4]. Thus, one of the major challenges in the management of patients with cGVHD is to optimize prevention of severe disease while maximising the graft-versus-leukaemia effect. Previous studies have demonstrated that mild cGVHD is associated with superior survival outcomes compared with non-cGVHD and moderate-severe cGVHD [5].

Currently, there are significant clinical limitations to fine-tuning this immunological balance. First, effective therapeutic options are scarce [1] and relatively ineffective. Corticosteroids, the standard first-line treatment for cGVHD [6], show sustained responses in only 40–50% of patients [7]. Secondly, there is both a lack of clinically available and reliable cGVHD biomarkers, preventing accurate stratification of patient risk, as well as a lack of standardized methods in clinical practice to accurately monitor patients’ immune function post-HSCT. Whilst this area has been and continues to be the subject of considerable study, even effective biomarker strategies [8] have not found their way into the clinic. This leads to potentially arbitrary adjustments of patient immunosuppression based on clinical experience rather than diagnostic standards. Prediction of GvHD development could permit personalized adjustments of immunosuppression modifications to ultimately prevent GvHD development or at least ameliorate its severity.

The 2005 National Institutes of Health (NIH) Consensus Conference established a framework to characterize cGVHD by standardising diagnostic criteria [9]. New guidelines were proposed to globally assess cGVHD severity and classification and these criteria were updated in 2014 [10]. However, an international survey has shown a limited uptake of these guidelines in clinical practice, [11] which is recognized by the NIH [12].

There is limited data on the burden of cGVHD from real-world studies; there are no central databases of patients with cGVHD diagnosed by NIH criteria, and there is a lack of complete data. As an integral part of Swedish healthcare, national registers contain comprehensive details of treatment and drug dispensation, along with inpatient and outpatient visits [13]. Similar to other countries, the disease burden of cGVHD and clinical outcomes associated with cGVHD have not been adequately assessed in Sweden. To address this unmet need, we used national registers to perform a retrospective, population-based longitudinal study. Instead of the NIH method based on the presentation of cGVHD, we used our newly introduced method [14] that considered the timing and extent of the immunosuppressive treatment given to HSCT recipients, with the objectives to investigate the burden of disease (including mortality and morbidities) and healthcare utilization in Sweden.

## Materials and methods

### National registers

Sweden has a universal, tax-funded healthcare system with equal access to all eligible residents. Eligible residents receive a unique personal identification number which enables longitudinal follow-up of individuals from birth to death. For the present study, four national population-based registers held by the National Board of Health and Welfare were linked: the Patient Register, the Cancer Register, the Cause of Death Register, and the Prescribed Drug Register. The diagnostic accuracy and completeness of the Swedish registers are high due to legally mandated reporting [15].

### Patient cohorts and definitions

**Fig 1:**
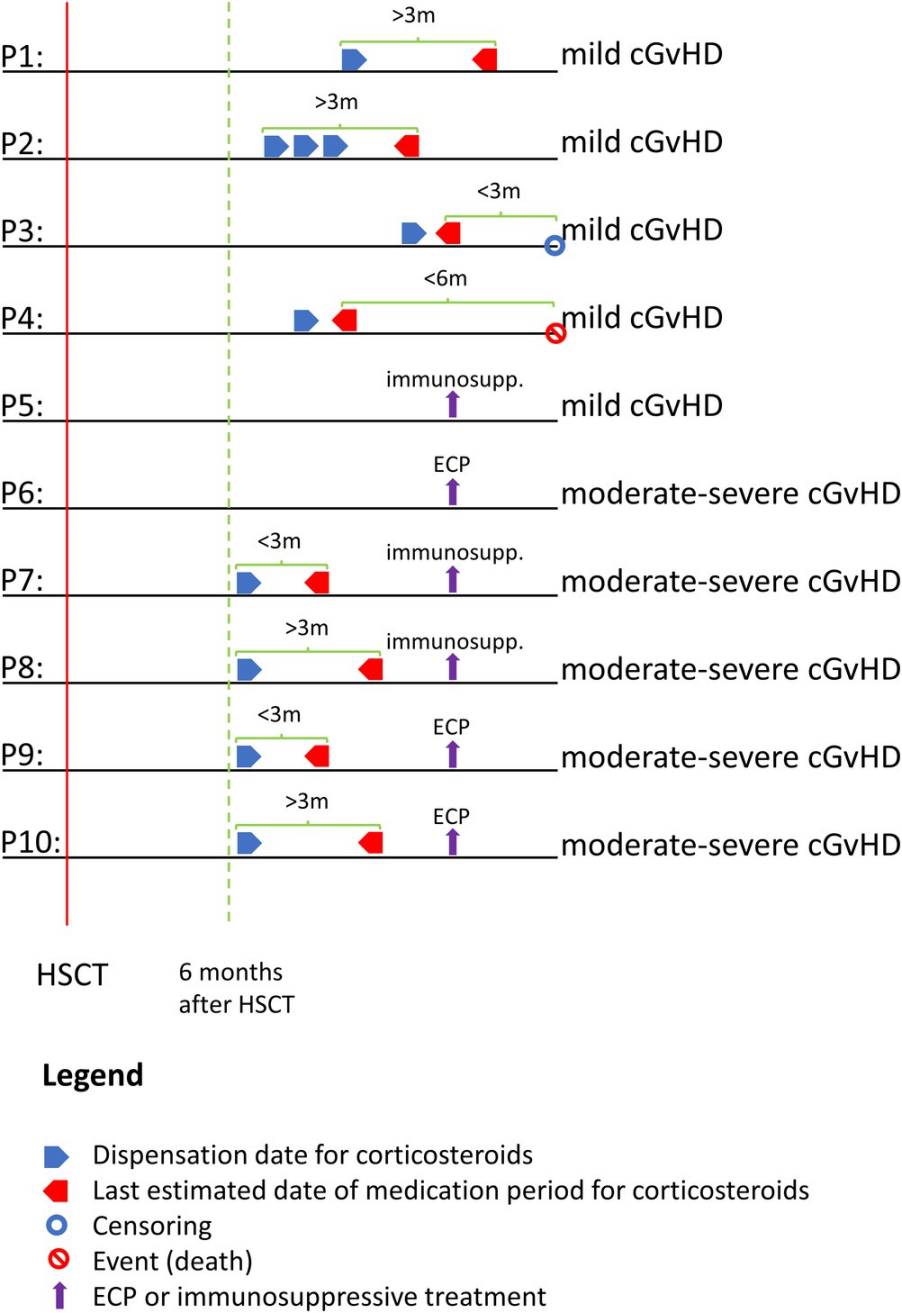
Definition of cGVHD status. Strategy used to retrospectively assign each patient who survived ≥6 months post-HSCT a cGVHD status based on the timing and extent to which patients received systemic immunosuppressive treatment. H02ABxx, glucocorticoids; immunosupp, immunosuppression; ECP, extracorporeal photopheresis; m, months; P, patient group. Fig 1 is adapted from Schain et al. [14].

All patients with a record of an allogeneic HSCT between 2006 and 2015 were identified in the Patient Register (n = 2147). This cohort and methods used for identification of cGVHD have been described previously [14]. Briefly, the following exclusion criteria were applied: absence of a haematological malignancy prior to the HSCT; reused proxy identification numbers; age <18 or >75; and death within 6 months post-HSCT (S1 Fig). cGVHD is commonly defined as occurring onwards of 6 months post-HSCT [16, 17]. For patients who survived ≥6 months post-HSCT (index date), the 0–6-month follow-up period was therefore 6–12 months post-HSCT. End of follow-up was Dec 31, 2016 (Cancer Register and Patient Register) and Dec 31, 2017 (Prescribed Drug Register and Cause of Death Register). Patient register records were reviewed, and patients were classified as having non-, mild, or moderate-severe cGVHD based on timing and extent of treatments commonly used for cGVHD using criteria developed by the authors (Fig 1) [14].

Patients were classified as non-cGVHD if, after taper of post-HSCT GvHD prophylaxis immunosuppression, they received neither systemic corticosteroids nor systemic immunosuppressive treatment (including everolimus, cyclosporine, methotrexate, mycophenolate, sirolimus, and tacrolimus) during the entire observation period. In Sweden, GvHD prophylaxis post-HSCT is discontinued by 3 months for matched sibling donors, and by 5 months for matched unrelated donors.

Patients with low-level cGVHD require less intensive immunosuppressive treatment. Based on this rationale, mild cGVHD was defined as patients receiving either corticosteroids or immunosuppressants alone. The following four mild cGVHD groups were defined: 1) patients who received systemic corticosteroid treatment >3 months alone, 2) patients whose last date of systemic corticosteroid treatment ended <3 months before censoring, 3) patients whose last date of systemic corticosteroid treatment ended <6 months before death, and 4) patients who received immunosuppressive treatment only (Fig 1).

Treatment modalities are similar for patients with moderate and severe cGVHD, which meant that it was not possible to separate these two groups. Patients with moderate-severe cGVHD require more intensive treatment than those with mild cGVHD. Based on this rationale, the following three moderate-severe cGVHD groups were defined: 1) patients who received corticosteroid treatment (irrespective of duration) and immunosuppressive treatment, 2) patients who received corticosteroid treatment (irrespective of duration) and extracorporeal photopheresis (ECP), and 3) patients who only received ECP.

Patients were assigned retrospectively to respective groups based on treatment (or not) received.

### Statistical analysis

All analyzes were performed based on patients who survived ≥6 months post-HSCT (index date). This was selected as it was more unlikely to include acute GVHD patients, however it may exclude some patients with true cGVHD who died early. Additionally, it may have included some patients who received donor lymphocyte infusion ≥ 6 months post-HSCT and developed acute GVHD. During the first 6 months post-HSCT, 193 patients died and were excluded from further analysis.

Chi-square and Kruskal–Wallis tests were used to compare the demographic and baseline differences between the three cGVHD groups. Overall survival (OS) rates were estimated for each group using the Kaplan-Meier method and the difference among the groups was analyzed by log-rank/Mantel–Haenszel test. The time-dependent hazard ratios (HRs) were estimated using a multivariate Cox-regression model to assess the relative effect of cGVHD on death rates, adjusting for age, sex, calendar year of HSCT, donor and stem cell source. Relapse-related mortality (RRM) and transplant-related mortality (TRM) were determined using data from the Cause of Death Register. When a haematological malignancy was stated as the main cause of a patient’s death, this case was considered for RRM. All other causes of death were considered for TRM.

Morbidities were classified using the International Statistical Classification of Diseases and Related Health Problems, 10th revision (ICD-10) codes. Morbidity crude incidence rates with 95% confidence intervals (CIs) were derived from a generalized linear Poisson univariate model for each complication. The adjusted incidence rate ratios (IRRs) and 95% CIs were obtained from a multivariate negative binomial model, adjusting for age, sex, and follow-up year with person-time at risk as offset.

Rate of healthcare resource utilization for each follow-up year was calculated as the total number of days in inpatient/outpatient care reported in the Patient Register for all causes, divided by the total days contributed to follow-up time for that follow-up year.

Data cleaning and statistical analyzes were performed using the statistical software R version 3.5.1 with the aid of R statistical package *survival*.

### Statement of ethics

Study approval was obtained from the Stockholm Ethical Review Board (dnr 2017-1716-31-1). The study has been granted an exemption from requiring written informed consent by the same ethical review board.

## Results

### Patient characteristics

We included 1246 patients in the final analysis who underwent HSCT from 2006 to 2015 and met our inclusion criteria. Of these patients, 28.1% were classified as non-cGVHD and 71.9% as cGVHD (27.7% as mild cGVHD, and 44.2% as moderate-severe cGVHD) (Table 1). Age, sex, year of HSCT, and source for HSCT were comparable among the three cohorts. However, an underlying diagnosis of acute leukemia was more common in patients classified as non-cGVHD (63%) compared to those who went on to experience moderate-severe (51%) and mild cGVHD (52%). Furthermore, matched related donor transplants were more common in patients who went on to experience moderate-severe cGVHD (38%) compared with non-cGVHD (27%) and mild cGVHD (26%) patients.

**Table 1:** Characteristics of Swedish patients who underwent HSCT between 2006 and 2015 and survived ≥6 months post-HSCT by cGVHD status.

	Non-cGVHD	Mild cGVHD	Moderate-Severe cGVHD	*P* value*	Overall
**Total (%)**	350 (28)	345 (28)	551 (44)		1246 (100)
**Sex, No. (%)**				0.618	
Men	206 (59)	192 (56)	323 (59)		721 (58)
Women	144 (41)	153 (44)	228 (41)		525 (42)
**Age at HSCT, No. (%)**				0.623	
18 to 39 years of age	92 (26)	79 (23)	132 (24)		303 (24)
40 to 59 years of age	147 (42)	164 (48)	256 (46)		567 (46)
60 to 75 years of age	111 (32)	102 (30)	163 (30)		376 (30)
Median age, years (Q1-Q3)	53 (41–61)	52 (40–61)	52 (39–62)	0.985	52 (40–61)
**Year of HSCT, No. (%)**				0.758	
2006 to 2010	116 (33)	122 (35)	195 (35)		433 (35)
2011 to 2015	234 (67)	223 (65)	356 (65)		813 (65)
**Diagnosis before HSCT, No. (%)**				0.002	
Acute leukemia	222 (63)	178 (52)	281 (51)		681 (55)
Lymphoma	31 (9)	54 (16)	64 (12)		149 (12)
Myelodysplastic syndrome	34 (10)	40 (12)	72 (13)		146 (12)
Chronic leukemia	27 (8)	45 (13)	75 (14)		147 (12)
Other hematologic malignancy	36 (10)	28 (8)	59 (11)		123 (10)
**Donor, No. (%)**				<0.0001	
Related	95 (27)	88 (26)	209 (38)		392 (31)
Unrelated	255 (73)	257 (74)	342 (62)		854 (69)
**Source for HSCT, No. (%)**				0.312	
Bone marrow/cord blood	76 (22)	91 (26)	126 (23)		293 (24)
PBSC	274 (78)	254 (74)	425 (77)		953 (76)

PBSC indicates peripheral blood stem cells; Q, quartile.

*P value for the univariate (Chi-square and Kruskal-Wallis) test of hypothesis of no difference between the three cGVHD groups.

### Overall survival

**Fig 2:**
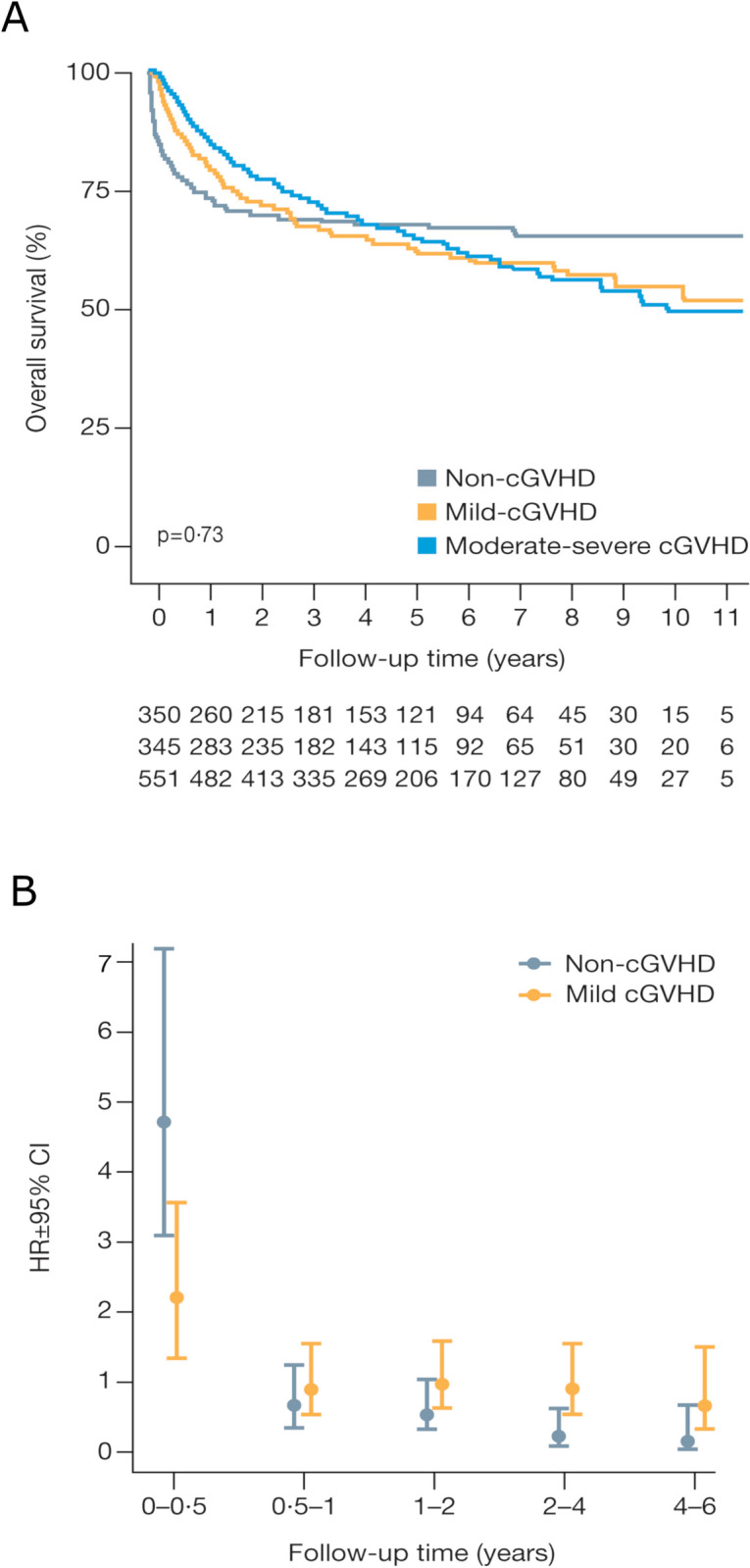
Overall survival rates and hazard ratios by cGVHD status among patients who survived ≥6 months post-HSCT. (A) Overall survival per follow-up year. (B) HRs and 95% CIs from the comparison of death rates in non-cGVHD and mild cGVHD patients, in reference to moderate-severe cGVHD patients, adjusted for age, sex, calendar year of HSCT, donor and source.

The median follow-up time for patients surviving ≥6 months post-HSCT was 3.5 years. The best OS at 1-year post-HSCT was in patients with moderate-severe cGVHD, which decreased with decreasing cGVHD severity status. Moderate-severe cGVHD patients had a 1-year OS rate of 87.5%, compared to 74.3% for non-cGVHD patients and 82.0% for mild cGVHD patients (P < 0.0001) (Fig 2A and Table 2). The non-cGvHD group experienced the lowest 1-year OS primarily due to relapse-related death. Overall survival was similar after 5 years of follow-up (non-cGVHD: 67.7%; mild cGVHD: 63.3%; moderate-severe cGVHD: 65.3%; P = 0.5) (Fig 2A and Table 2). OS during the entire follow-up period did not significantly differ (P = 0.73) among the three patient groups. In all patient groups, more patients died from RRM than TRM (Table 2 and S2 Fig).

**Table 2:** Relapse-related mortality, transplant-related mortality, median follow-up time, and 1- and 5-Year OS rates by cGVHD status among patients who survived ≥6 months post-HSCT.

	Non-cGVHD (n = 350)	Mild cGVHD (n = 345)	Moderate-Severe cGVHD (n = 551)	*P* value	Overall (n = 1246)
**Deceased, No. (%)**				0.009*	
**RRM**	98 (28)	109 (32)	147 (27)		354 (28)
**TRM**	16 (5)	21 (6)	57 (10)		94 (8)
**Survived**	236 (67)	215 (62)	347 (63)		798 (64)
**Median follow-up time, years (Q1-Q3)**	3.2 (0.9–6.2)	3.3 (1.6–6.3)	3.9 (2.0–6.7)		3.5 (1.6–6.4)
**1-year OS rate (95% CI)**	74 (70–79)	82 (78–86)	88 (85–90)	<0.0001^*§*^	82 (80–84)
**5-year OS rate (95% CI)**	68 (63–73)	63 (58–69)	65 (61–70)	0.500^*§*^	65 (63–68)

Q indicates quartile.

*P value for the univariate (Chi-square and Kruskal-Wallis) test of hypothesis of no difference between the three cGVHD groups.

^*§*^*P* value produced from the Mantel-Cox log-rank test.

### Mortality hazard ratios

In patients who survived the first 6 months post-HSCT, multivariate analysis was adjusted for sex, age, calendar period, donor source, and donor relationship. Multivariate and univariate analysis revealed that during the 0- to 6-month follow-up period (6–12 months post-HSCT), the risk of death was significantly higher in non- and mild cGVHD than in moderate-severe cGVHD patients (Fig 2B and Table 3). There was no evidence for HSCT treatment period, sex or donor relationship having an impact on the risk of death (P > 0.05) when analyzing the full follow-up period using multivariate and univariate analyzes (Table 3). However, patients aged 60 to 75 years who underwent HSCT had an increased HR for mortality compared with patients aged 18 to 39 years. This equates to a 42% and 47% greater risk of mortality, by univariate and multivariate analyzes, respectively (Table 3). In addition, compared with patients who received bone marrow or cord blood as a donor source, patients who received peripheral blood stem cells (PBSCs) showed a reduction in HR for mortality, equating to 22% and 23% by univariate and multivariate analyzes, respectively.

**Table 3:** Hazard ratios for death of patients who survived ≥6 months post-HSCT.

	Univariate analysis HR (95% CI)	*P* value* (univariate)	Multivariate analysis HR (95% CI)	*P* value* (multivariate)
**cGVHD** ^**a**^				
Moderate-severe cGVHD	1.00 (ref)		1.00 (ref)	
Non-cGVHD	4.73 (3.08–7.25)	<0.0001	4.72 (3.07–7.24)	<0.0001
Mild cGVHD	2.26 (1.40–3.65)	0.001	2.21 (1.37–3.56)	0.001
**Sex** ^b^				
Male	1.00 (ref)		1.00 (ref)	
Female	0.98 (0.81–1.18)	0.842	0.97 (0.81–1.18)	0.791
**Age at follow-up, years** ^b^				
18–39	1.00 (ref)		1.00 (ref)	
40–59	1.17 (0.92–1.49)	0.206	1.21 (0.95–1.54)	0.132
60–75	1.42 (1.10–1.83)	0.008	1.47 (1.14–1.91)	0.004
**Calendar year at the start of follow-up** ^b^				
2006–2010	1.00 (ref)		1.00 (ref)	
2011–2015	0.89 (0.73–1.09)	0.258	0.86 (0.71–1.06)	0.150
**Donor** ^b^				
Related	1.00 (ref)		1.00 (ref)	
Unrelated	1.11 (0.91–1.35)	0.322	1.10 (0.90–1.36)	0.348
**Source for HSCT** ^b^				
Bone marrow/cord blood	1.00 (ref)		1.00 (ref)	
Peripheral blood stem cells	0.78 (0.63–0.95)	0.01–6	0.77 (0.63–0.95)	0.016

*ref* indicates reference group.

^a^Reported hazard ratio estimates are from the index dates (≥6 months post-HSCT) and 6 months forward. Hazard ratios for later follow-up timepoints are presented in Fig 2B.

^b^Hazard ratios are calculated for the full follow-up period.

^*^P value produced using a univariate and multivariate Cox-regression model, respectively.

### Morbidities

After adjusting for age, sex, and follow-up year, the IRRs for morbidities were assessed across all disease categories in the ICD system (Table 4). The incidence of most morbidities was significantly higher among moderate-severe cGVHD patients (p<0.05) versus non- (21–83% higher IRRs) and mild cGVHD patients (10–77% higher IRRs) (Table 4). Significantly higher morbidity rates were observed in moderate-severe cGVHD patients in all but two categories compared with non-cGVHD patients, and all but three categories compared with mild cGVHD patients (Table 4). Moderate-severe cGVHD patients reported 83% and 77% higher rate ratios of injury, poisoning, and certain other consequences of external causes (ICD-10 S00-T98) versus non- and mild cGVHD patients, respectively. A confounding factor is that ICD category S00-T98 includes the code for GVHD.

**Table 4:** Incidence of morbidities by cGVHD status among patients who survived ≥6 months post-HSCT.

	Crude incidence rates per 1000-person years (95% CI)			Adjusted incidence rate ratios (95% CI)	
Description of Disease/Disorder	Non-cGVHD	Mild cGVHD	Moderate-Severe cGVHD	Non-cGVHD *vs* Moderate-Severe cGVHD	Mild cGVHD *vs* Moderate-Severe cGVHD
Certain infectious and parasitic diseases	215 (191–242)	167 (146–190)	243 (224–264)	0.76 (0.52–1.03)	0.69 (0.53–0.91)
Endocrine, nutritional and metabolic diseases	63 (51–78)	140 (121–162)	158 (143–175)	0.41 (0.22–0.75)	0.90 (0.47–1.41)
Mental, behavioural, and neurodevelopmental disorders	40 (30–52)	95 (80–113)	112 (99–126)	0.36 (0.14–0.74)	0.74 (0.30–1.64)
Diseases of the nervous system	26 (18–36)	31 (23–42)	60 (51–71)	0.44 (0.27–0.68)	0.50 (0.31–0.76)
Diseases of the eye and adjacent organs	230 (205–258)	341 (311–374)	705 (672–739)	0.32 (0.23–0.42)	0.49 (0.34–0.68)
Diseases of the ear and mastoid process	48 (37–62)	33 (25–45)	61 (51–71)	0.79 (0.40–1.25)	0.48 (0.28–0.74)
Diseases of the circulatory system	67 (54–83)	100 (84–119)	177 (160–194)	0.32 (0.22–0.47)	0.53 (0.36–0.78)
Diseases of the respiratory system	152 (132–175)	167 (146–190)	272 (252–294)	0.49 (0.36–0.63)	0.59 (0.45–0.75)
Diseases of the digestive system	86 (71–103)	117 (100–137)	229 (211–249)	0.39 (0.27–0.52)	0.49 (0.35–0.65)
Diseases of the cutaneous and subcutaneous tissue	112 (95–132)	93 (78–111)	148 (133–164)	0.73 (0.58–0.94)	0.60 (0.45–0.77)
Diseases of the musculoskeletal system and connective tissue	91 (76–109)	140 (121–161)	236 (217–256)	0.39 (0.24–0.55)	0.62 (0.44–0.84)
Diseases of the genitourinary system	196 (173–222)	231 (206–258)	255 (235–276)	0.75 (0.59–0.94)	0.83 (0.63–1.05)
Injury, poisoning, and certain other consequences of external causes^a^	155 (134–177)	255 (229–284)	1001 (961–1041)	0.17 (0.11–0.23)	0.23 (0.18–0.29)

Morbidities are categorised by International Classification of Diseases code. Incidences within the disease group for the same month are counted separately. Generalised linear Poisson model was used with person-years as offset.

^a^This class (S00-T98) also includes GVHD.

### Healthcare resource utilization

**Fig 3:**
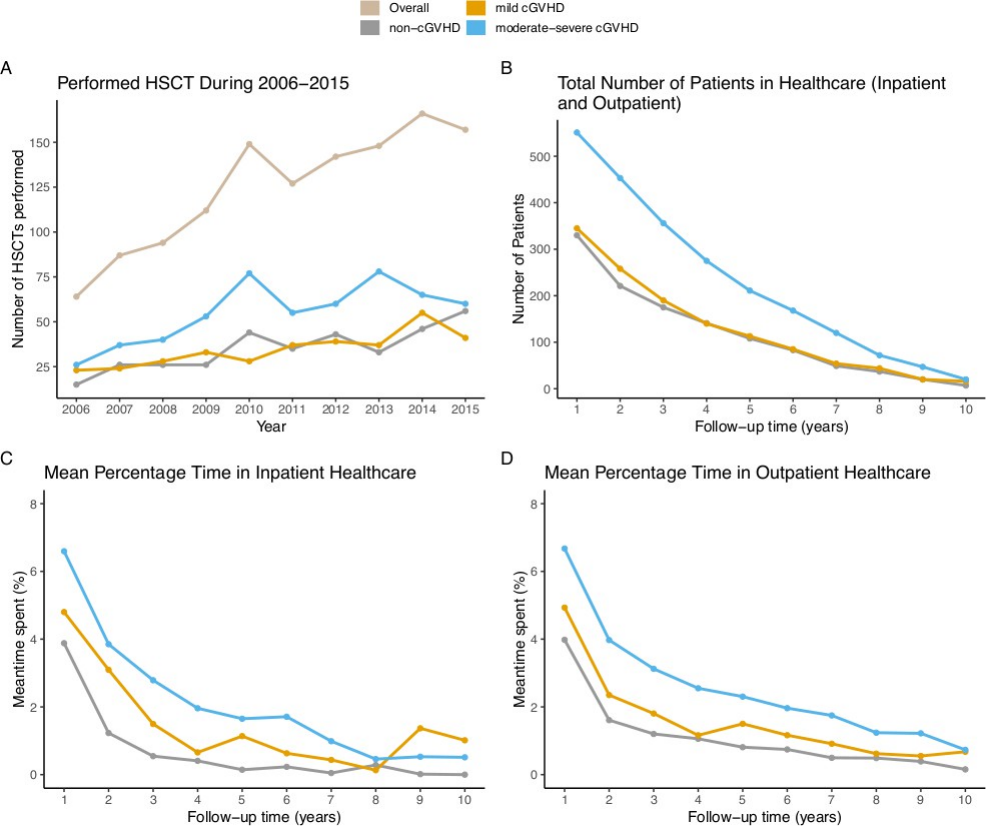
Healthcare resource utilization by cGVHD status among patients who survived ≥6 months post-HSCT. (A) Number of HSCTs performed during 2006–2015. (B) Total number of patients in healthcare (inpatient and outpatient) per follow-up year. (C) Average proportion of time patients spent in inpatient healthcare per follow-up year. (D) Average proportion of time patients spent in outpatient healthcare per follow-up year.

We determined the clinical burden associated with cGVHD in terms of healthcare utilization in patients who survived ≥6 months post-HSCT. Those patients who did not survive to ≥ 6 months post-HSCT (n = 193) were excluded. The number of transplants more than doubled during the 10-year study period, while the proportion of patients with cGVHD stayed approximately constant (Fig 3A and Table 1). More patients having moderate-severe cGVHD following HSCT received inpatient or outpatient healthcare than non- or mild cGVHD throughout the whole follow-up period (Fig 3B). In total, the proportion of moderate-severe cGVHD patients using healthcare at 1-year follow-up was about 1.6-fold higher compared to non- or mild cGVHD patients (Fig 3B). Furthermore, moderate-severe cGVHD patients spent a larger proportion of their follow-up time in inpatient healthcare compared with non- and mild cGVHD patients (equating to 59% and 31% more, respectively) (Fig 3C); similarly, this group spent a larger proportion of their follow-up time in outpatient healthcare compared with non- and mild cGVHD patients (equating to 54% and 36% more, respectively) (Fig 3D).

## Discussion

Allogeneic HSCT is a potential cure for patients with haematological malignancies [18]. While treatment has improved significantly over time [19], HSCT remains a complex treatment, where fine-tuning of the immunological responses is needed and is controlled by various immunosuppressive strategies before and after HSCT. The debilitating and long-term impact of cGVHD in a large proportion of patients who undergo HSCT is well-established, yet there is a paucity of studies addressing the real-world incidence of cGVHD. In our previous study, we demonstrated a significantly higher incidence of cGVHD than previously reported [14]. Through the use of Swedish health registers that allow for patient-level linkage across registers and that provide population-wide data without selection bias [20], we now provide a detailed analysis of clinical outcomes of cGVHD patients including survival, morbidities, and healthcare utilization. Using our recently introduced real-world treatment-based criteria [14], we were able to more accurately identify patients that were treated for cGVHD, a diagnosis which is underreported due to low adoption of standardized criteria [21].

The NIH recommendations are the standard criteria used to assess cGVHD although there is a limited uptake in clinical practice (lack of adherence has been reported to be as high as 60%) [11]. A recent report by the NIH has recognized that while the current diagnostic and severity criteria are well established, transplant providers struggle with their implementation, and providers with less experience with chronic GVHD, such as primary oncologists or other clinicians who sometimes resume the care of patients after HSCT, may be even less adept at recognizing the earliest symptoms and signs [12]. Thus, there is potential for variable and incorrect diagnoses. Due to uncertainty around adherence to NIH criteria, in this study cGVHD status was assigned retrospectively based on the timing and extent to which patients actually received the standard of care therapies for cGVHD. This approach demonstrated an estimated incidence of cGVHD of 71.9% in patients who survived at least 6 months post-HSCT; considerably higher than previously reported using the standard NIH criteria in Sweden (38%) [21]. The incidence of cGVHD reported internationally varies dramatically from 6% to 80%, but 50% has been considered a reasonable estimate [17]. One possible reason for this discrepancy may be reporting bias, as physicians may not always report cGVHD when they put patients on appropriate immunosuppressive treatment.

In the current study we have shown that 6–12 months post-HSCT, death rate was higher in the non- and mild cGVHD group compared with the moderate-severe cGVHD group. Strikingly, the non-cGVHD patients who survived ≥6 months post-HSCT had a 4.72 times higher death risk at 6 months of follow-up, compared with those with moderate-severe cGVHD. This suggests a negative clinical impact of over-immunosuppression through GvHD prophylaxis (e.g. T-cell depletion, duration of post-HSCT calcineurin inhibitor prophylaxis), and that a more personalized approach is required, balancing disease relapse and cGvHD risks. Furthermore, the 1-year overall survival rate improved with increasing cGVHD severity. This improved survival is supported by a systematic review which also showed that patients with cGVHD had higher overall survival compared with those patients with malignancy without cGVHD [22]. Patients with related donors experienced more moderate-severe cGvHD which may be due to the practice of avoiding T-cell depletion in this group of patients, due to the expected reduced allo-reactivity from closer minor and major HLA-matching in sibling donor-recipient pairs [21].

To avoid including patients with acute GVHD, a cut-off 6 months post-HSCT was used. This is the most accepted time frame for the resolution of acute GVHD and the onset of cGVHD. In support of this, others have shown that some clinicians utilizing consensus criteria have historically considered any GvHD (including acute) to be cGvHD if it occurred after 100 days [23]. However, a cut-off 6 months post-HSCT may have not excluded those few cases developing acute GVHD following donor lymphocyte infusion. Nevertheless, this 6-month cut-off post-HSCT excluded a high proportion of patients with RRM from our analysis. Regardless of this, RRM was higher than TRM in all cGVHD groups; meaning that more patients died from relapse than treatment. Most morbidities assessed occurred significantly more often in moderate-severe cGVHD patients versus those with non- and mild cGVHD. As previously reported, the incidence or prevalence of diseases of the eye, digestive system disease, cutaneous and subcutaneous tissue, and musculoskeletal system morbidities are all considered direct manifestations of cGVHD [24–27]. In the present study, moderate-severe cGVHD patients had significantly higher rates of most ICD categories, compared with non-cGVHD patients. However, it is possible that ICD codes may have been inconsistently applied [28], which is supportive of the methodology used in the current study using immunosuppressive therapy to characterize patients with cGvHD. Limitations using the current methodology (where cGvHD was diagnosed by inference) should be noted given that some patients may not have been correctly categorized or been excluded: exclusion of mild NIH cGVHD cases from cGVHD since these do not typically receive systemic immunosuppression, potential loss of cGVHD cases which presented <6 months after transplant, potential inclusion of late aGVHD in cGVHD diagnosis, and the inability to separate NIH moderate from NIH severe cases. Importantly the role of the methodology described in the current study allows for a different perspective to account for cGvHD detection, using registries for population-based resources utilization analyzes in long term survivors after allogeneic HSCT. However, this method may not be applicable for more detailed and clinically or biologically meaningful inferences about cGVHD.

Higher rates of morbidities translated into greater healthcare utilization for moderate-severe cGVHD patients. This is evident from the increased proportion of time that moderate-severe cGVHD patients spent in inpatient and outpatient healthcare compared with non- and mild cGVHD patients, highlighting the impact of cGVHD on healthcare resources as well as patient quality of life. We have previously shown that the cumulative total costs during the first 3 years of follow-up post HSCT were EUR 14,887,599, EUR 20,544,056, and EUR 47,811,835 for non-, mild, and moderate–severe groups cGVHD, respectively [14].

Although our method to classify cGVHD, as opposed to the established NIH criteria is novel, it highlights an urgent need for standardized methods to monitor immune function post-HSCT in clinical practice. The necessary tools required to prospectively identify specific patients at risk are currently lacking. To date most research has focused on early identification of patients at risk of developing severe cGVHD with little attention directed at identifying patients who may be over-immunosuppressed. More research should now be directed at this non-cGVHD group who have a significantly increased risk of death during the first year after allogeneic HSCT. Efficient methods are therefore needed to monitor immune function that will enable data-driven treatment decisions to be made in real time. Once these have been identified and implemented, prospective trials can be initiated to validate the observations. Until then, clinical outcomes for patients with non- and moderate-severe cGVHD will remain inferior to patients with mild cGVHD, posing a considerable strain on healthcare delivery.

Our registry-based method for identifying patients with cGVHD may not be fully concordant with the NIH cGVHD classification. However, the Prescribed Drug Register in Sweden has previously been used to define disease severity [29, 30]. Importantly, validation studies demonstrated a high degree of sensitivity and positive predictive value when compared with the established methods used in clinical trials [31, 32]. Moreover, compared with non-cGVHD patients, the higher proportion of TRM observed in cGVHD patients, and the higher rates of morbidities and healthcare utilization for moderate-severe cGVHD support the accuracy of our registry-based method. Whilst it might have been more customary to use the NIH classification to determine cGvHD organ involvement and severity, we sought to demonstrate the feasibility of studying the real-world incidence of cGvHD from a different perspective, where we previously demonstrated a significantly higher incidence of cGVHD than previously reported [14]. In contrast to the previous study, which was focused on healthcare utilization, we aimed to examine in more detail the clinical outcomes of those patients, with the advantage of being able to capture all the patients receiving treatment for moderate-severe cGvHD. Thus, this approach may capture some patients being treated by non-HSCT specialists in regional hospitals and hence may not be reported to HSCT unit patient databases. On the other hand, there may also be included in the moderate-severe cGvHD group (determined by those requiring systemic treatment) some patients with mild cGvHD who were on tapering immunosuppression for other reasons than cGvHD or related to a resolving episode of acute GvHD, thus including patients with potentially less TRM and better outcome in the moderate-severe cGvHD groups. However, we anticipate that, again, this number will be small.

In conclusion, our population-based registry study indicates that cGVHD is underreported in Sweden. Patients without cGVHD had significantly increased death rates in the first year post-trantsplant, possibly due to the intensity or duration of GvHD prophylaxis, and suggesting that a more personalized approach balancing relapse risk and cGvHD risk is required. Patients with moderate-severe cGVHD had higher rates of morbidities and healthcare resource utilization versus patients with non- and mild cGVHD. We conclude that to improve patient outcomes, validated clinically approved and standardized assays to measure immune function are needed, as well as novel treatments that better balance the competing requirements of immune suppression and optimisation of graft-versus-leukaemia effects.

## Supporting information

S1 Fig Selection of patient cohort who survived ≥3 months and ≥6 months post-HSCT. (TIF)

S2 Fig Cumulative incidence of transplant-related mortality (TRM) and relapse-related mortality (RRM) by chronic graft-versus-host disease (cGVHD) status. Non-cGVHD (A), mild cGVHD (B) and moderate-severe cGVHD patients (C), who survived ≥3 months post-HSCT. Non-cGVHD (D), mild cGVHD (E) and moderate-severe cGVHD (F) who survived ≥6 months post-HSCT.
(TIF)

S1 File Inclusivity in global research. (DOCX)
