# Supplementary figures and images for: Population-based real-world registry study to evaluate clinical outcomes of chronic graft-versus-host disease
Source: PLoS One. 2023 Mar 9;18(3):e0282753. doi: 10.1371/journal.pone.0282753 (PMC9997892; doi:10.1371/journal.pone.0282753)

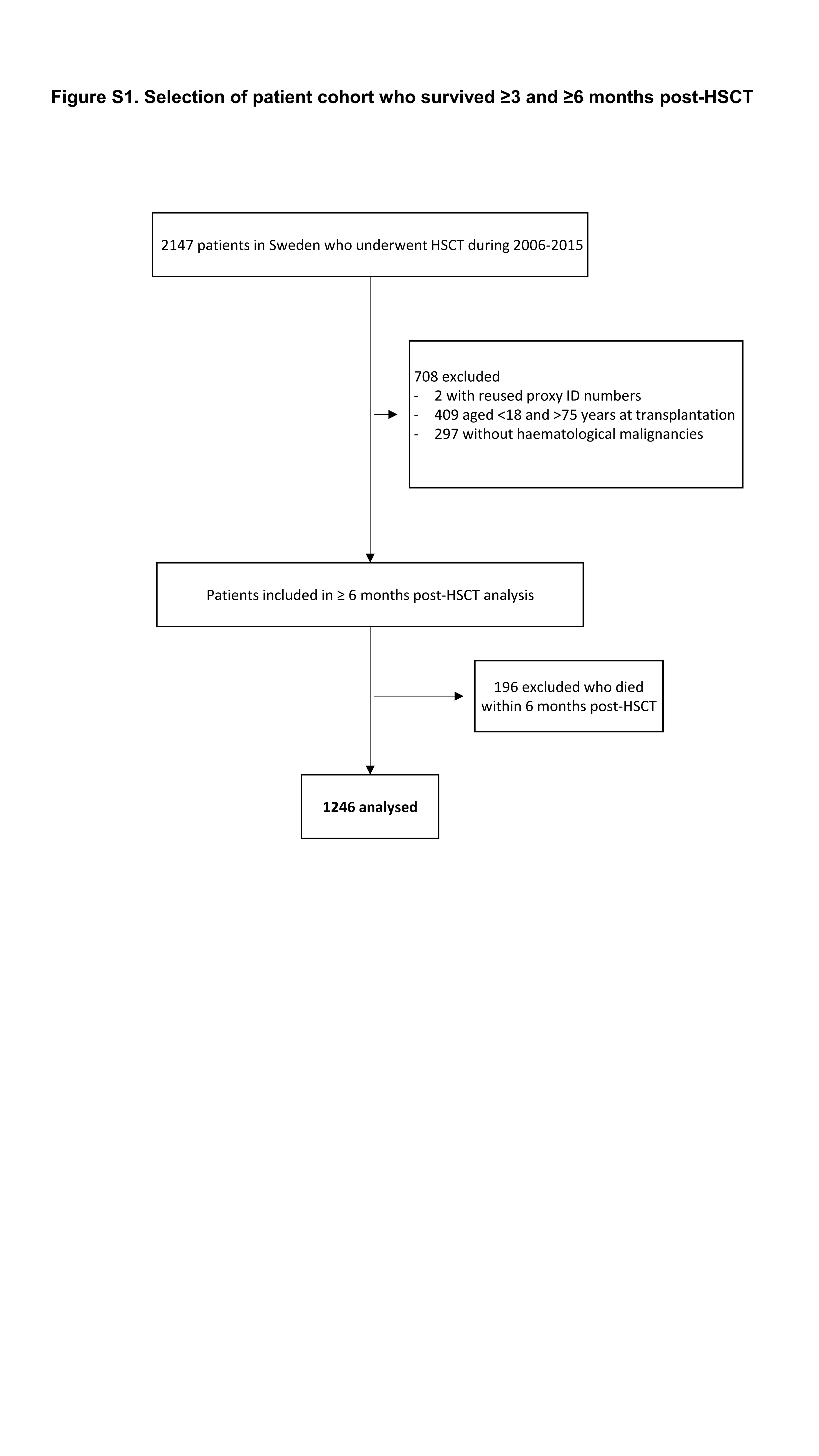

Supplement: S1 Fig — (TIF) [file pone.0282753.s001.tif]

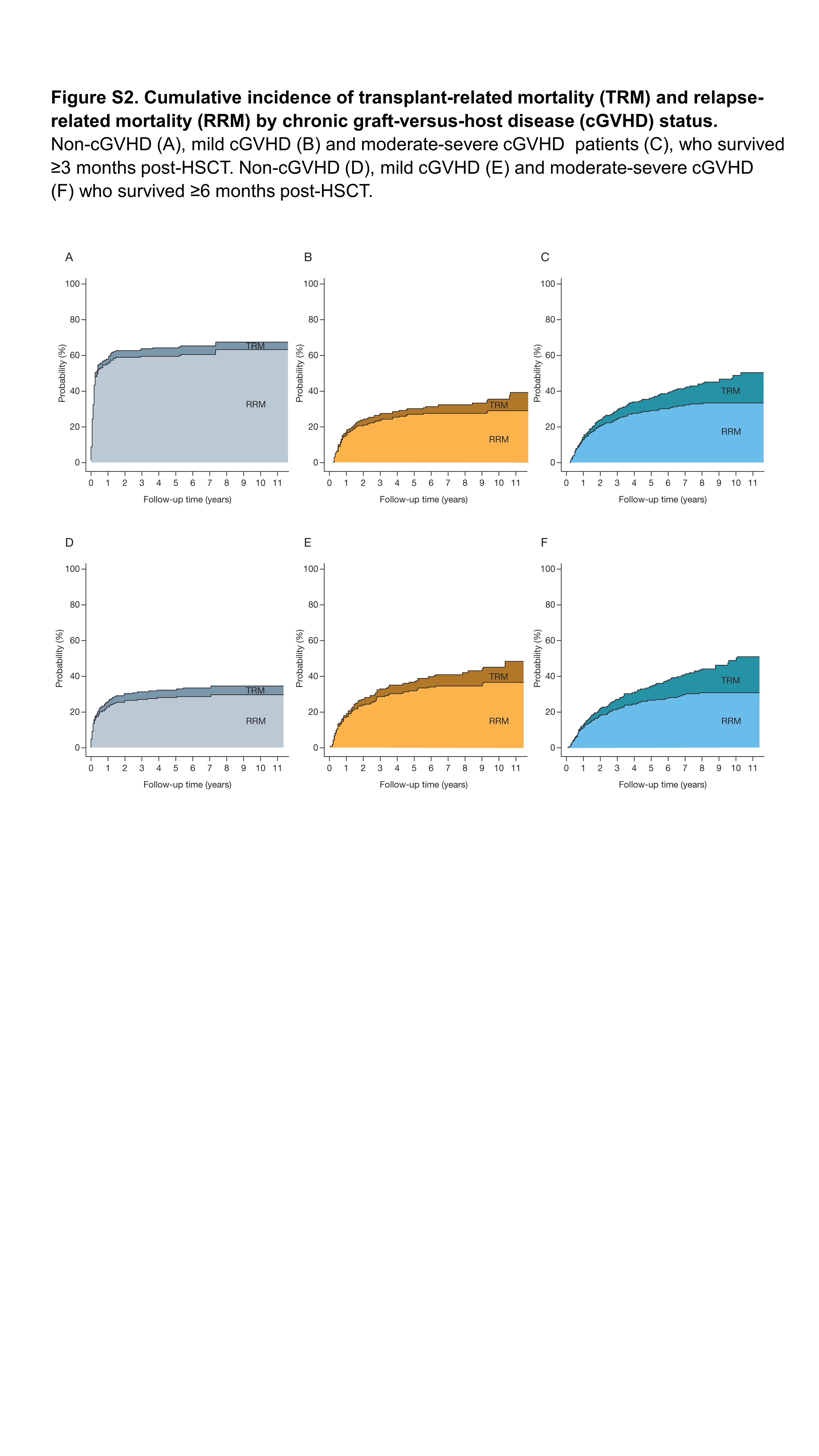

Supplement: S2 Fig — Non-cGVHD (A), mild cGVHD (B) and moderate-severe cGVHD patients (C), who survived ≥3 months post-HSCT. Non-cGVHD (D), mild cGVHD (E) and moderate-severe cGVHD (F) who survived ≥6 months post-HSCT. (TIF) [file pone.0282753.s002.tif]
